# Antitumor activity of curcumin is involved in down-regulation of YAP/TAZ expression in pancreatic cancer cells

**DOI:** 10.18632/oncotarget.12596

**Published:** 2016-10-12

**Authors:** Xiuxia Zhou, Jingna Su, Shaoyan Feng, Lixia Wang, Xuyuan Yin, Jingzhe Yan, Zhiwei Wang

**Affiliations:** ^1^ The Cyrus Tang Hematology Center and Collaborative Innovation Center of Hematology, Jiangsu Institute of Hematology, The First Affiliated Hospital, Soochow University, Suzhou 215123, China; ^2^ Department of Otolaryngology, The fifth Affiliated Hospital of Sun Yat-sen University, Zhuhai, 519020, China; ^3^ Department of Abdominal Oncosurgery, Jilin province Cancer Hospital, Changchun, Jilin, 130012, China; ^4^ Department of Pathology, Beth Israel Deaconess Medical Center, Harvard Medical School, Boston, MA 02215, USA

**Keywords:** curcumin, pancreatic cancer, Skp2, invasion, proliferation

## Abstract

Pancreatic cancer (PC) is one of the most aggressive human malignancies worldwide and is the fourth leading cause of cancer-related deaths. Curcumin (diferuloylmethane) is a polyphenol derived from the *Curcuma longa* plant. Certain studies have demonstrated that curcumin exerts its anti-tumor function in a variety of human cancers including PC, via targeting multiple therapeutically important cancer signaling pathways. However, the detailed molecular mechanisms are not fully understood. Two transcriptional co-activators, YAP (Yes-associated protein) and its close paralog TAZ (transcriptional coactivator with PDZ-binding motif) exert oncogenic activities in various cancers. Therefore, in this study we aimed to determine the molecular basis of curcumin-induced cell proliferation inhibition in PC cells. First, we detected the anti-tumor effects of curcumin on PC cell lines using CTG assay, Flow cytometry, clonogenic assay, wound healing assay and Transwell invasion assay. We found that curcumin significantly suppressed cell growth, weakened clonogenic potential, inhibited migration and invasion, and induced apoptosis and cell cycle arrest in PC cells. We further measured that overexpression of YAP enhanced cell proliferation and abrogated the cytotoxic effects of curcumin on PC cells. Moreover, we found that curcumin markedly down-regulated YAP and TAZ expression and subsequently suppressed Notch-1 expression. Collectively, these findings suggest that pharmacological inhibition of YAP and TAZ activity may be a promising anticancer strategy for the treatment of PC patients.

## INTRODUCTION

Pancreatic cancer (PC) is a highly aggressive human malignancy worldwide with an extremely poor prognosis [1]. For the majority of the PC patients, the only therapeutic promise is cytostatic treatment using standard chemotherapeutic drugs such as gemcitabine and 5-FU (5-fluorouracil) or their combination [2]. However, the median survival time of PC patients is only about six months mostly due to an almost complete chemotherapy resistance, and the dismal 5-year survival is currently approximately 2% [3]. Therefore, there is a dire need for both new biomarkers with prognostic and predictive value and newer therapeutic options for this disease.

Natural edible products could be important therapeutic agents for the treatment of a lot of human diseases including cancer. Among these agents, curcumin, a naturally occurring polyphenolic compound of turmeric, has several pharmacologic properties under both pre-clinical and clinical conditions [4]. Curcumin exhibits its anticancer effects against different types of cancer by targeting multiple therapeutically important cancer signaling pathways such as Ras, mTOR (mammalian target of rapamycin), FOXO1 (forkhead box protein 1), Wnt/β-catenin, PI3K (phosphoinosmde-3-kinase) and AKT pathways [5–8]. These results revealed that targeting numerous of signaling molecules regulated by curcumin could represent a novel strategy for the treatment of PC patients.

Two homologous transcriptional co-activators, YAP (yes-associated protein) and TAZ (transcriptional coactivator with PDZ-binding motif), known as key downstream effectors of Hippo signaling pathway, play a key role in organ growth control, stem cell function, and tissue development and regeneration [9–14]. Upon defects in Hippo signaling or other stimuli, YAP and TAZ translocate to nucleus and bind to transcription factors (e.g., TEAD1-4 (TEA domain family member 1-4), ErbB4(receptor tyrosine protein kinase erbB-4), SMAD (mother against decapentaplegic homolog 4), and p73), promote expression of genes that drive cell proliferation and inhibit cell death [12, 15, 16]. In recent years, YAP and TAZ have drawn intense attention for their remarkable biological properties in cancers [17, 18]. YAP and TAZ are deregulated in various human malignancies [16, 19–22]. Lysine methyltransferase SETD7 (SET domain containing lysine methyltransferase 7)-dependent methylation of YAP facilitates Wnt-driven intestinal tumorigenesis and regeneration [22]. Activation of TAZ is thought as a major event in breast cancer initiation and/or progression, [23]. Over-expression of TAZ is directly associated with mesenchymal differentiation and high-grade tumors in human malignant glioma and breast cancer [23, 24].

In this study, we examined whether YAP and TAZ were targets of curcumin in PC cells. We also determined whether YAP and TAZ were involved in the anti-proliferative function of curcumin. Here, we present evidence that curcumin induced cell growth inhibition and apoptosis of PC cells. YAP and TAZ were down-regulated after curcumin treatment. Moreover, Notch signaling was activated by YAP over-expression and restrained by YAP inhibition. Our results suggested that inhibition of YAP/TAZ and Notch signaling by curcumin could be considered for treatment of advanced PC.

## RESULTS

### The cytotoxic effects of curcumin on PC cells

**Figure 1:**
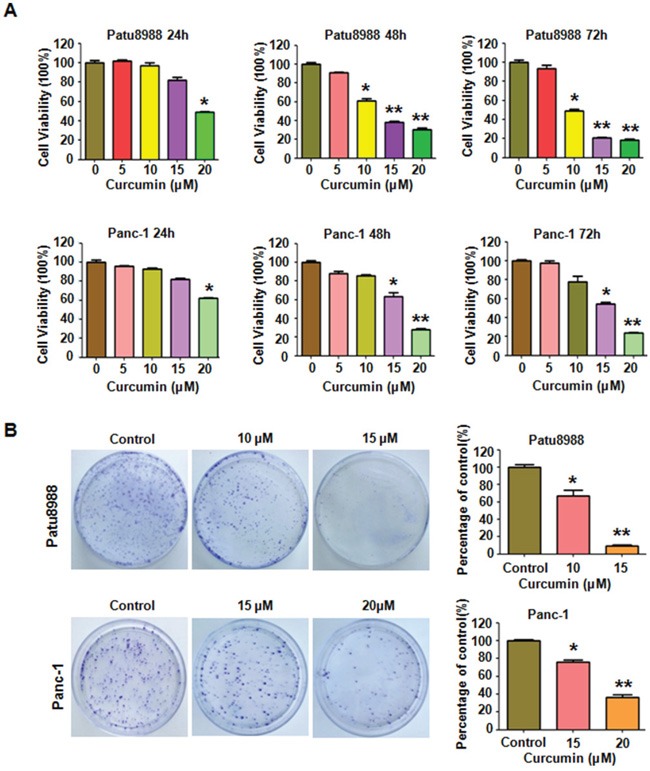
Curcumin inhibited PC cells growth **A.** Effect of curcumin on PC cells growth was detected by CTG assay after treatment with curcumin for 24, 48 and 72h, respectively. *P < 0.05, **P < 0.01, compared to the control groups (DMSO treatment group). **B.** Clonogenic assay was performed to evaluate the colony formation viability of PC cells treated with curcumin. *P < 0.05, **P < 0.01 vs control.

In order to explore the potential cytotoxic effect of curcumin on PC cells, CTG assays and colony formation assays were performed. Patu8988 and Panc-1 cells were treated with various concentrations of curcumin for 24, 48 and 72 hours, respectively. CTG assays showed that curcumin clearly reduced the growth viability of PC cells in the time- and dose-dependent manners in comparison with the control (Figure 1A). The half maximal inhibitory concentrations (IC50) of curcumin for Patu8988 and Panc-1 cells at 72 hours were found to around 10μM and 15 μM, respectively (Figure 1A). Next, the colony-forming ability of curcumin-treated PC cells was investigated. The results showed a significant decrease in colony formation upon curcumin treatment in a dose-dependent manner (Figure 1B). Overall, in alignment with CTG data, the results from colonogenic assay indicated the cytotoxic effects of curcumin on PC cells.

### Curcumin induced apoptosis of PC cells

**Figure 2:**
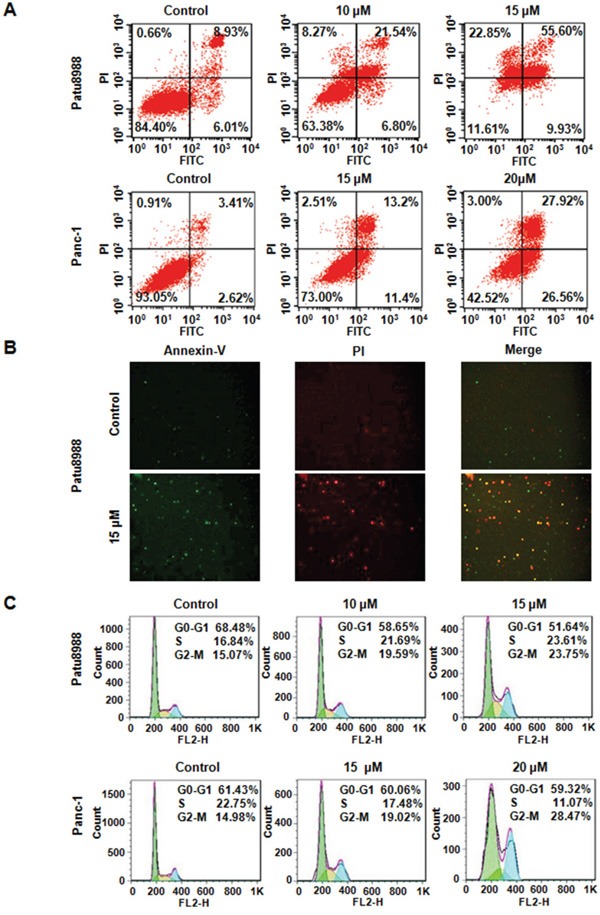
Curcumin induced PC cells apoptosis and caused cell cycle arrest **A.** Cell apoptosis in curcumin-treated PC cells was accessed by Flow cytometry. Control: DMSO treatment. **B.** Apoptotic PC cells were stained with FITC-labeled Annexin V (green fluorescence) and PI (red fluorescence) and observed using a fluorescence microscope. **C.** Curcumin induced PC cell cycle arrest at G2-M Phase.

Evidences had showed that curcumin-mediated cell growth inhibition could be attributed to the increased apoptosis. To investigate whether curcumin triggered apoptosis in PC cells, an apoptotic assay was employed. PC cells were stained with FITC-labeled Annexin V (green fluorescence) and PI (red fluorescence) for apoptosis detection. As shown in Figure 2A, Annexin V-FITC/PI apoptosis flow cytometric detection showed that curcumin treatment triggered cell apoptosis in both Patu8988 and Panc-1 cells (Figure 2A). The apoptosis rates were increased from 14.94% of control group to 28.34% and 65.53% of 10 and 15 μM curcumin-treated Patu8988 cells (Figure 2A). Simultaneously, both Annexin V and PI positive staining was observed in curcumin-treated Patu8988 cells (Figure 2B). Similar increased apoptosis rates were also observed in Panc-1 cells (Figure 2A). These results clearly indicated that curcumin significantly triggered apoptosis of PC cells, and this apoptotic effect was exerted in a dose-dependent manner.

### Curcumin induced cell cycle arrest

To investigate whether curcumin could abrogate cell cycle progression in PC cells, cell cycle distribution after PI staining was analyzed in both Patu8988 and Panc-1 cells treated with curcumin for 48 hours. We identified an increased accumulation of the cell population in the G2/M phase from 15.07% with control to 19.59% and 23.75% with 10 and 15 μM curcumin treatments in Patu8988 cells (Figure 2C). Similarly, curcumin treatment caused a typical G2/M arrest pattern in Panc-1 cells in a dose-dependent manner (Figure 2C). These findings showed that curcumin treatment could distinctly lead to a G2/M phase arrest in PC cells.

### Curcumin inhibited cell migration and invasion

**Figure 3:**
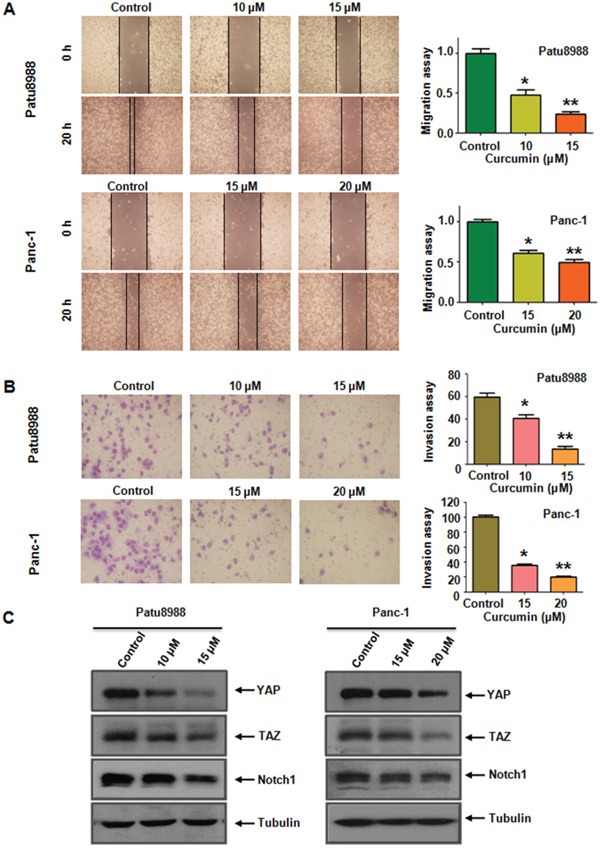
Curcumin inhibited cell migration and invasion in PC cells and inhibited YAP/TAZ expression **A.** The inhibitory effect of curcumin on PC cell migration was detected using wound healing assay in Patu8988 cells (upper, left panel) and Panc-1 cells (under, left panel). Right panel, quantitative results are illustrated for left panels. *P < 0.05, **P < 0.01, vs control group (DMSO treatment). **B.** The inhibitory effect of curcumin on PC cell invasion was detected by Transwell chambers assay in Patu8988 cells (upper, left panel) and Panc-1 cells (under, left panel). Right panel, quantitative results are illustrated for left panel. *P < 0.05, **P < 0.01 vs control. **C.** Curcumin inhibited YAP/TAZ and Notch-1 expression at protein levels in Patu8988 cells (left panel) and Panc-1 cells (right panel).

We evaluated the effect of curcumin on PC cell motility using wound-healing assays and Transwell assay, as the capacity of cancer cell migration is considered one of the critical processes in the development of tumor metastasis. Wound-healing assay demonstrated that curcumin treatment significantly led to reduced wound closure in both PC cells at the 20-hour time point (Figure 3A). In accordance with this, curcumin treatment resulted in a decreased penetration of PC cells that could invade the matrigel-coated chamber (Figure 3B). Moreover, curcumin inhibited PC cells motility in a dose-dependent manner (Figure 3B). Taken together, curcumin indeed exerts its inhibitory effect on PC cell motility.

### Curcumin decreased YAP/TAZ expression

YAP/TAZ has been documented to be involved in tumorigenesis. Therefore, we focus on the alterations in the cell survival pathway with special emphasis on YAP/TAZ to further explore the underlying molecular mechanism of the oncogenic functions of curcumin in PC cells. Western blotting analysis revealed that YAP and TAZ levels were significantly down-regulated in both PC cells after curcumin treatment compared with controls (Figure 3C and Supplementary Figure S1). Emerging evidences showed that Notch signaling intersected with YAP/TAZ in cancer progression and tumorigenesis. Therefore, we detected the expression of Notch-1 in PC cells with curcumin treatment. We found a significant inhibitory effect of curcumin on Notch-1 expression. Curcumin-induced down-regulation of YAP/TAZ is involved in the decrease of Notch-1. Altogether, curcumin exerts its anti-cancer property via down-regulation of YAP/TAZ and Notch signaling pathway.

### Over-expression of YAP promoted cell proliferation and reversed the effects of curcumin on PC cells

**Figure 4:**
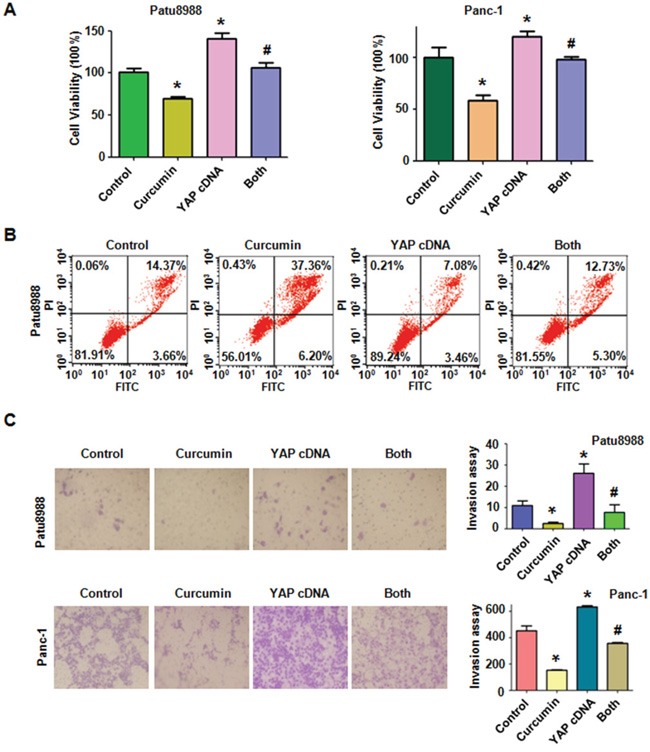
Overexpression of YAP triggered cell proliferation, abrogated cell apoptosis and promoted cell invasion in PC cells **A.** The effect of YAP overexpression in combination with curcumin treatment on PC cell growth was detected by CTG assay. Control: pcDNA3.1 transfection; Both: YAP cDNA+Curcumin. *P < 0.05, compared with control; # P < 0.05 compared with curcumin treatment or YAP cDNA transfection. **B.** Cell apoptosis was accessed by Flow cytometry. **C.** Cell invasion was detected by Transwell chambers assay. *P < 0.05, compared with control; # P < 0.05 compared with curcumin treatment or YAP cDNA transfection.

In order to determine whether curcumin exerts its anti-tumor activity partly through down-regulation of YAP/TAZ in PC cells, Patu8988 cells and Panc-1 cells were transfected with YAP cDNA or empty vector as control. We found that over-expression of YAP promoted PC cells growth and partly abrogated curcumin-induced cell growth inhibition (Figure 4A). We then detected whether transfection of YAP cDNA could reverse curcumin-induced apoptosis. As expected, over-expressed YAP indeed significantly reduced percentage of apoptotic cells in Patu8988 cells (Figure 4B). Importantly, over-expression of YAP reversed curcumin-induced apoptosis in Patu8988 cells, which suggested that the apoptosis induced by curcumin treatment could be partly due to down-regulation of YAP/TAZ. Moreover, we performed Transwell assay to verify the contribution of YAP to the invasion potential of PC cell lines. We found that over-expression of YAP enhanced the invasion abilities in both Patu8988 and Panc-1 cells (Figure 4C). Notably, over-expression of YAP abrogated the inhibitory effects of curcumin on cells motility.

**Figure 5:**
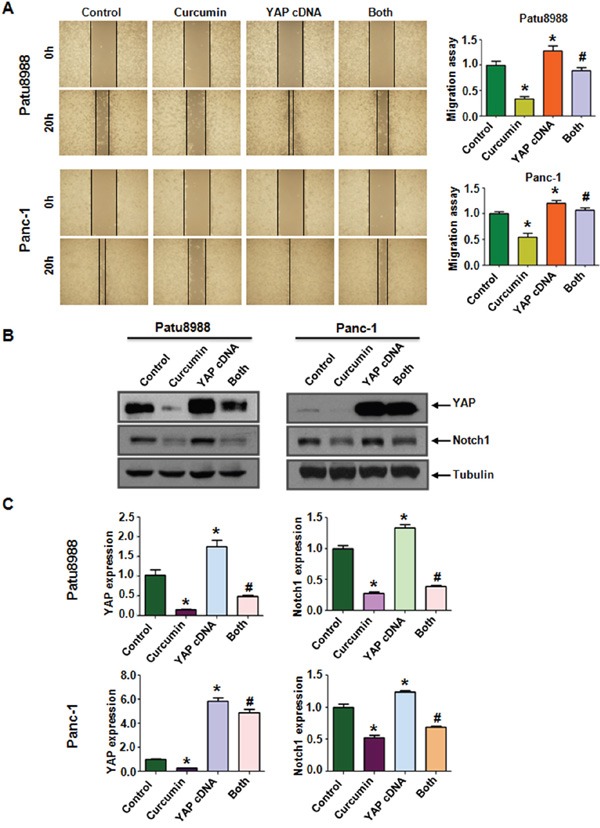
Overexpression of YAP enhanced PC cells migration **A.** Left panel, PC cells migration after YAP cDNA transfection and curcumin treatment was detected by wound healing assay. Control: pcDNA3.1 transfection; Both: YAP cDNA+Curcumin. Right panel, Quantitative results are illustrated for left panel. **B.** The expression of YAP and Notch-1 was measured in YAP cDNA-transfected PC cells treated with curcumin. **C.** Quantitative results were illustrated for panel B. *P < 0.05, compared with control; # P < 0.05 compared with curcumin treatment or YAP cDNA transfection.

Next, wound healing assay was performed to verify the contribution of YAP to the migration potential of PC cell lines. Enhanced migration abilities were observed in both PC cell lines tranfected with YAP cDNA (Figure 5A). Over-expression of YAP attenuated the inhibitory effects of curcumin on cells migration abilities. We further measured the downstream targets of YAP following YAP cDNA transfection. The results showed that over-expression of YAP activated its downstream target Notch-1 (Figure 5B and 5C). Moreover, over-expression of YAP reversed the inhibitory effect of curcumin on Notch-1 to a certain degree. These results suggest that curcumin exerts its anticancer function partially by inactivation of YAP/TAZ and Notch-1 signaling in PC cells.

### Down-regulation of YAP by siRNA transfection sensitized PC cells to curcumin

**Figure 6:**
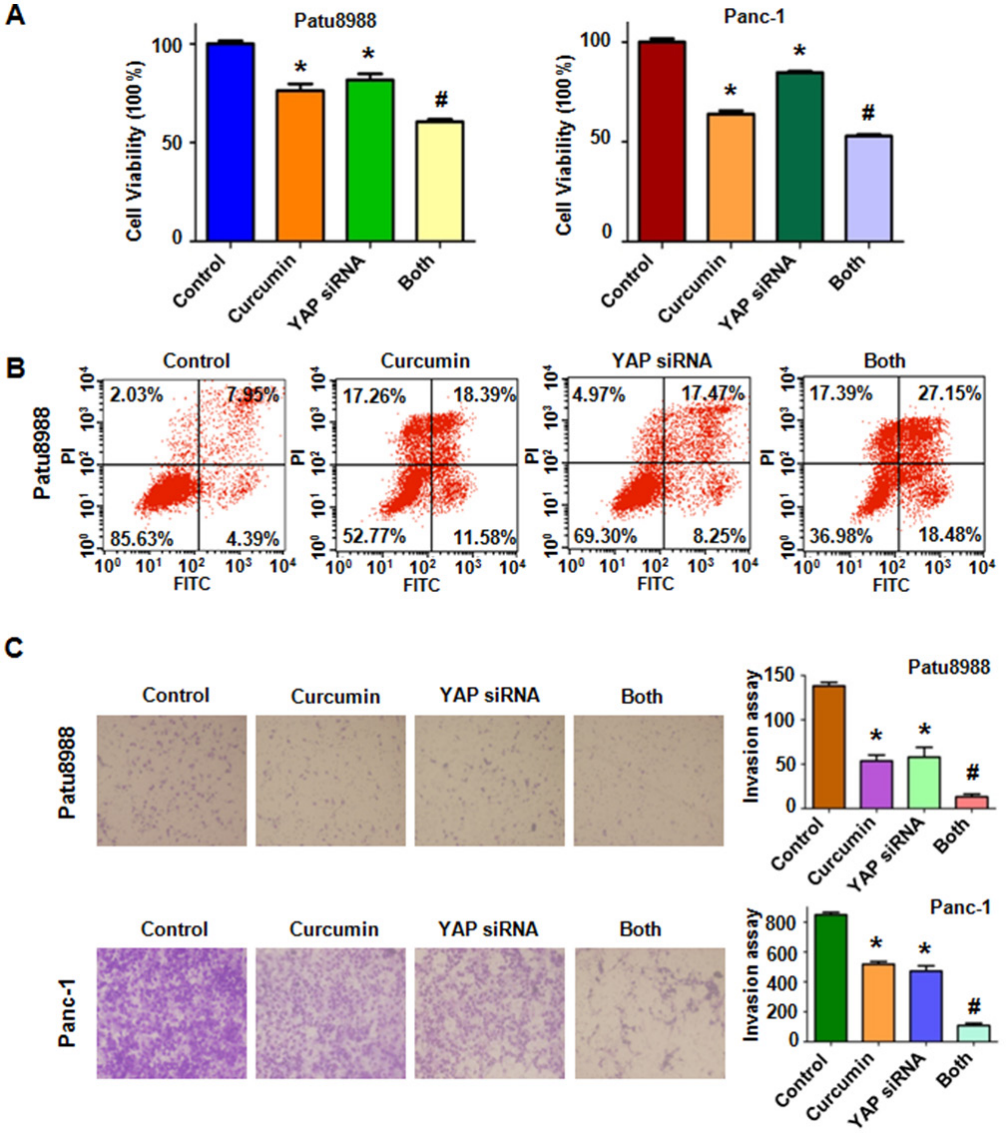
Knockdown of YAP inhibited cell proliferation, promoted cell apoptosis and inhibited cell invasion in PC cells **A.** The effect of down-regulated YAP in combination with curcumin treatment on PC cell growth was detected by CTG assay. Control: non-specific control siRNA; Both: YAP siRNA+Curcumin. **B.** Cell apoptosis was accessed by Flow cytometry. **C.** Cell invasion was detected by Transwell chambers assay. *P < 0.05, compared with control; # P < 0.05 compared with curcumin treatment or YAP siRNA transfection.

**Figure 7:**
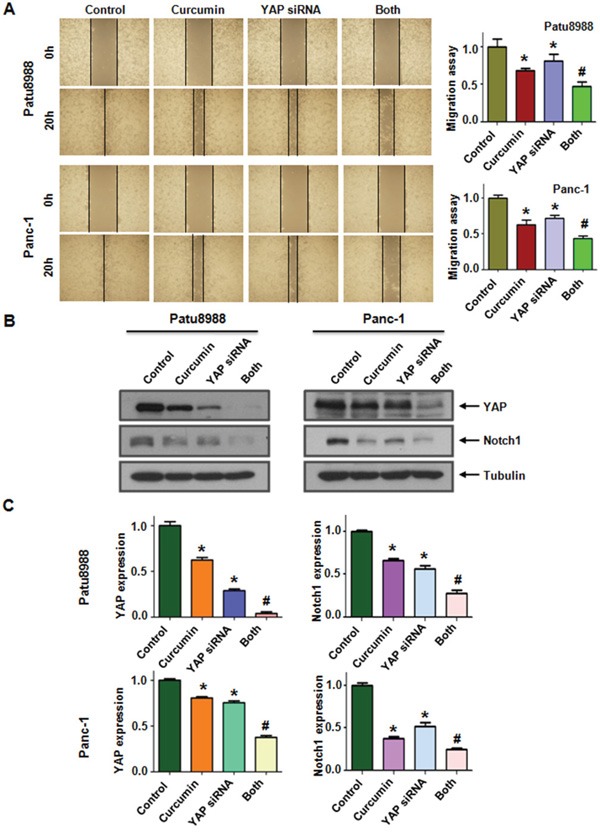
Knockdown of YAP inhibited PC cells migration **A.** Left panel, PC cells migration after YAP siRNA transfection and curcumin treatment was detected by wound healing assay. Control: non-specific control siRNA; Both: YAP siRNA+Curcumin. Right panel, Quantitative results are illustrated for left panel. **B.** The expression of YAP and Notch-1 was measured in YAP siRNA-transfected PC cells treated with curcumin. **C.** Quantitative results were illustrated for panel B. *P < 0.05, compared with control; # P < 0.05 compared with curcumin treatment or YAP siRNA transfection.

We performed viability assays on Patu8988 and Panc-1 cells again to further explore whether the modulation of the YAP/TAZ-Notch-1 signaling axis would ultimately lead to cellular susceptibilities to the cytotoxicity of curcumin. Both PC cells were transfected with YAP siRNA oligonucleotides. The results showed that depletion of YAP markedly inhibited cell growth (Figure 6A). Combined with curcumin, down-regulation of YAP enhanced cell growth inhibition to a greater degree compared with curcumin alone or siRNA transfection alone. Moreover, we found that Patu8988 cells were significantly more sensitive to spontaneous and curcumin-induced apoptosis upon YAP down-regulation (Figure 6B). Further, we identified that down-regulation of YAP notably suppressed invasion and migration in PC cells (Figure 6C and 7A). Finally, we also found a decreased expression of Notch-1 via YAP depletion (Figure 7B and 7C). More importantly, curcumin plus YAP siRNA inhibited Notch-1 activity to more degree compared to curcumin alone or siRNA transfection alone (Figure 7B and 7C). Collectively, these results indicated that concomitant with curcumin and YAP siRNA not only synergistically enhanced curcumin-induced cytotoxicity in both cell lines but also rendered PC cells more susceptible to curcumin.

## DISCUSSION

In the present study, we showed the cytotoxic effects of curcumin on PC cell lines. We found that curcumin significantly suppressed cell proliferation and promoted cell apoptosis in a dose-dependent manner in both Patu8988 and Panc-1 cells. Next, we demonstrated that curcumin induced a typical G2/M phase arrest in both PC cells. We further identified an evident inhibition of cell migration and invasion in curcumin treated PC cells. Moreover, down-regulation of YAP and TAZ proteins were observed in PC cells treated with curcumin. Mechanistically, Notch-1, one of YAP/TAZ targets, was also suppressed, suggesting an intersection of YAP/TAZ and Notch signaling might contribute to the cytotoxic effects of curcumin on PC cells.

Multiple studies have underscored the importance of YAP/TAZ in gastrointestinal tissue tumorigenesis, including PC. YAP and TAZ are present in normal pancreatic centroacinar and ductal cells, and are upregulated in cancer cells. In particular, YAP and TAZ are expressed at high levels in the activated stellate cells of pancreatic ductal adenocarcinoma patients [25]. These findings supported a critical role of YAP and TAZ in modulating pathogenesis of pancreatic diseases. The human adenocarcinoma-associated gene, AGR2 (anterior gradient 2), induces expression of amphiregulin through activation of YAP1 in PC [26]. Zhang and his colleague demonstrated that YAP1 was identified as a critical and functional downstream which plays a role in the oncogenic switch between K-RAS pathway and MAPK (mitogen activated protein kinase) pathway in PC [27]. A recent study reveals that activated YAP interacted with TEAD2 and E2F transcription factors, leading to bypass of oncogenic Kras addiction in PC [28]. Jagged-1 and Notch-2 were identified as direct YAP–TEAD target genes when YAP was ectopic activated in the adult liver. Hes-1, one of Notch targets, was up-regulated due to the hepatocyte-specific over-expression of YAP [29]. Additionally, increased abundance of total YAP and pancreatic progenitor cells markers—Hes-1 and Sox-9—were observed in the mutant pancreata of Mst1/Mst2 double knockout mice [30]. YAP1 and TAZ controlled PC initiation in mice by direct up-regulation of JAK–STAT3 signaling [31]. Noteworthy, emerging data showed that YAP1 is negatively regulated by miR-141 [32], miR-375 [33] and miR-181c [34], which serves an independent prognostic factor for PC patients and functions as tumor suppressors. Moreover, miR-181c directly repressed MST1 (mammalian STE20-like protein kinase 1), LATS2 (large tumor suppressor 2), MOB1 (MOB kinase activator 1) and SAV1 (Salvador homologue 1), leading to YAP/TAZ activation and subsequent promotion of PC cell survival and chemoresistance both *in vitro* and *in vivo* [34]. YAP/TAZ functions as a signaling nexus and integrator of several other prominent signaling pathways, suggesting that pharmacological inhibition of YAP and TAZ activity may provide an effective anticancer strategy.

Small-molecule inhibitors and activators of Hippo signaling have been identified by cell based high throughput screening. Actually, more than 100 compounds were identified from a screen of approximately 3300 FDA (food and drug administration) approved drugs for inhibitors of the nuclear localization and transcriptional activity of YAP [35]. Among these inhibitors, dobutamine was identified to prevent nuclear accumulation of YAP and YAP-mediated transcriptional activation in osteoblastoma and HEK293 cells [36]. Verteporfin (VP) was found to bind to YAP *in vitro* and to inhibit the interaction of YAP with TEAD [35]. And VP was effective in delaying tumor progression in a NF2-depleted mouse liver model. VP also suppressed liver overgrowth caused by over-expression of YAP in this model. However, future studies will be needed to determine whether these drugs are effective in other cancer models. More importantly, efforts will be made to determine whether these compounds are effective in the treatment of established cancers. Additionally, the affinity of these compounds for YAP/TAZ should be considered.

Curcumin was reported to exhibit its anticancer effects against different types of cancer, including PC, by targeting multiple therapeutically important cancer signaling pathways. Curcumin promoted KLF5 (krueppel-like 5) proteasome degradation via down-regulating YAP/TAZ in bladder cancer cells [21]. Previous study had demonstrated that curcumin-induced down-regulation of Notch-1 is associated with the inhibition of cell growth in lung cancer cells [37]. Noteworthy, in contract with other cytotoxic drugs, curcumin has minimal toxicity and is safety at high dose by human clinical trials [38, 39]. Therefore, suppression of YAP/TAZ and Notch signaling by curcumin could provide a promising therapeutic strategy for the treatment of PC patients. However, therapeutic use of curcumin is hampered due to its rapid metabolism and poor absorption [40]. Undoubtedly, both aggrandize the bioavailable efficiency and/or improve delivery methods of curcumin are required to overcome the blood-brain barrier in therapeutic use. In addition, further studies will be necessary to determine detailed mechanism which curcumin exerts its anti-cancer function through inhibiting YAP/TAZ and Notch signaling in PC.

## MATERIALS AND METHODS

### Cell culture

The PC cell lines Patu8988 and Panc-1 were maintained in GIBCO^®^DMEM (Thermo Fisher Scientific, USA) supplemented with 10% FBS (HyClone, USA) and 1% Penicillin-Streptomycin (Thermo Fisher Scientific, USA) in a 5% CO2 atmophere at 37°C.

### Cell viability assay

The Patu8988 and Panc-1 cells (4×10^3^) were seeded in a 96-well plate. After an overnight culture, cells were treated with different concentrations of curcumin for 48 h and 72 h. Curcumin (CAS number 458-37-7, 99.5% purity) was obtained from Sigma-Aldrich (St. Louis, MO). Cells were treated with 0.1% DMSO as the control group. CellTiter-Glo Luminescent Cell Viability Assay (CTG, Promega) was carried out by following the manufacture's instruction. Independent experiments were repeated in triplicate.

### Clonogenic assay

3×10^5^ per well Patu8988 and Panc-1 cells were plated in 6-well plates and incubated overnight. After about 72 h exposures to different concentrations of curcumin, the viable cells were collected and counted. 3,000 collected PC cells were seeded into a 100 mm dish and subsequently incubated for 21 days at 37°C in a humidified 5% CO2 atmosphere. All the colonies were stained with 2% crystal violet to examine the survival of cells treated with curcumin.

### Cell apoptosis analysis

Patu8988 and Panc-1 cells were first seeded at a density of 3 × 10^5^ cells/well in 6-well plates and allowed to incubate at 37°C overnight. After treatment with various concentrations of curcumin for the indicated time intervals, cells were trypsinized and harvested by centrifugation. The collected cells were washed with PBS, and resuspended in 500 μl of binding buffer containing 5 μl Propidium iodide (PI) and 5 μl annexin V-FITC for 15 min under dark conditions. Then, all of the samples were analyzed immediately using a FACS calibur flow cytometer (BD, USA) to detect the apoptosis induced by curcumin treatment. Curcumin triggered apoptosis of PC cells were also observed under a fluorescence microscope.

### Cell cycle analysis

To determine the effect of curcumin on the cell cycle, PC cells were seeded at a density of 3×10^5^ cells/well in 6-well titer plates and incubated at 37°C overnight. Then, the cells were exposed to indicated concentration of curcumin for 48h. Cells were collected and fixed with ice-cold 70% (v/v) ethanol and kept at 4°C overnight. Thereafter, cells were collected and washed with PBS. The cell pellets were re-suspended and stained in PBS containing 0.1mg/ml RNase I and 50 mg/ml PI for 30 min at room temperature. Cell distribution across the cell cycle was determined with a FACScalibur flow cytometer (BD, USA).

### Wound healing assay

Patu8988 and Panc-1 cells were seeded in 6-well plates at the concentration of 2×10^6^ cells per well and incubated at 37°C overnight. Cell monolayers that converged almost 100% were wounded with a sterile 100 μl pipette tip. Remove detached cells from the plates carefully with PBS and add DMEM. PC cells were left either untreated or stimulated with the indicated doses of curcumin. After the incubation for 20h, medium was replaced with PBS and the scratched areas were photographed using an Olympus microscope.

### Transwell invasion assay

Cell invasive capacity was determined using a Transwell chamber (8μm pore size, Corning) with Matrigel (BD Biosciences) according to the manufacturer's instructions. Briefly, suitable amount of PC cells treated with curcumin or YAP transfection or combination were placed on each upper chamber in 200 μL of serum-free DMEM plus 0.1% DMSO or transfection control. In the lower chamber, 500 μL of complete medium (containing10% FBS) was added with the same concentration of curcumin. After 24h of incubation at 37°C in 5% CO_2_, cells in the upper surface of the membranes were removed. The cells that had migrated through the pores and attached on the underside of the membrane were stained with Wright's-Giemsa. At least six randomly-selected images were counted and the average number of stained cells represented the relative invasion.

### Transfection

PC cells (3 ×10^5^ cells/well) were grown in 6-well plates, exposed to certain concentration of curcumin and transfected with YAP cDNA or YAP siRNA or empty vector using lipofectamine 2000 according to the manufacturer's instructions. YAP siRNA oligonucleotides were purchased from GenePharma (Shanghai, China): sense 5′-GGU GAU ACU AUC AAC CAA ATT-3′; antisense 5′-UUU GGU UGA UAG UAU CAC CTT-3′.

### Western blotting analysis

Cells were harvested and lysed in cell lysis buffer (Cell Signaling Technology, Danvers, MA, USA), quantified and heated for 5 min at 100°C. Equal amount of denatured protein samples were decentralized on a SDS-polyacrylamide gel and then transferred onto a PVDF membrane. Specific primary antibody was added to the membrane and then incubated at 4°C overnight. After washed 3 times with TBST, the membrane was then incubated with horseradish peroxidase–conjugated second antibody at room temperature for 1 hour. The protein bands were subsequently visualised using ECL reagents (Pierce, Rockford, IL, USA). Antibodies against YAP/TAZ (recognizes endogenous levels of total YAP and TAZ) and Notch1 (recognizes intracellular domain of Notch1) were purchased from Cell Signaling Technology (Danvers, MA, USA). The membranes were stripped with 0.2M NaOH and reprobed with tubulin primary antibody (Sigma-Aldrich, St. Louis, MO, USA) as the loading control. ImageJ software was used to calculate the ensitometric quantification of the bands. The results were presented as fold change relative to the control after normalization with tubulin.

### Statistical analysis

All data analyses were expressed as mean ± SD of triplicates. Statistical analysis of data was conducted using GraphPad Prism 4.0 (Graph Pad Software, La Jolla, CA). Differences between each group of values and its control group were evaluated by the 2-tailed Student's t test and differences with a p<0.05 were considered significant.

## SUPPLEMENTARY FIGURES AND TABLES


