# Supplementary material for: Antitumor activity of curcumin is involved in down-regulation of YAP/TAZ expression in pancreatic cancer cells
Source: Oncotarget. 2016 Oct 12;7(48):79076–88. doi: 10.18632/oncotarget.12596 (PMC5346699; doi:10.18632/oncotarget.12596)
Supplement: Supplementary file 1 [file oncotarget-07-79076-s001.pdf]

Antitumor activity of curcumin is involved in down-regulation of YAP/TAZ expression in pancreatic cancer cells

SUPPLEMENTARY FIGURE

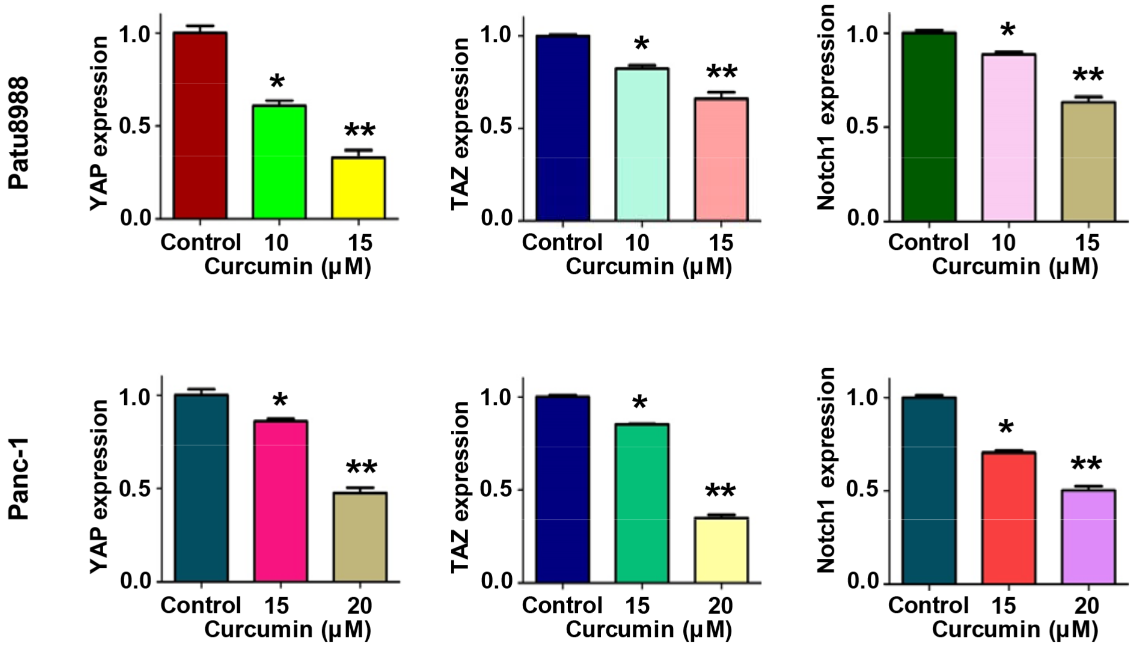

Supplementary Figure S1: Quantitative results are illustrated for Figure 3C.
